# Relaxing the Clock Brings Time Back into Phylogenetics

**DOI:** 10.1371/journal.pbio.0040106

**Published:** 2006-03-14

**Authors:** Richard Robinson

The hypothesis of a “molecular clock”—a constant rate of mutation over evolutionary time—revolutionized phylogenetics, the study of evolutionary relationships among organisms. Using the assumption of this constant rate, one can determine the time since two organisms diverged from a common ancestor simply by toting up the number of DNA sequence differences between them. Thus, the molecular clock provided an important tool for constructing phylogenies, “trees” of relatedness, for organisms as diverse as primates and protists.

However, the constancy of the mutation rate, both between different groups and within a single group over time, has been repeatedly challenged. As a result, the molecular clock has been largely abandoned in recent years for constructing phylogenetic trees. In its place has arisen a model that accepts that each branch may have its own rate of mutation. Relatedness between two organisms can still be determined and trees can still be drawn, but without a constant mutation rate, no estimate can be made of the time since divergence, and thus the position in time of the last common ancestor—the “root” of the tree—cannot be calculated.

An alternative approach, termed a “relaxed molecular clock,” has been developed to overcome the difficulties of both the molecular clock and unrooted phylogeny models. In a new study, Alexei Drummond, Andrew Rambaut, and colleagues describe a new approach to relaxed-clock analysis, showing that it can be used to simultaneously construct accurate trees and infer times of divergence.

Previous attempts to reintroduce a molecular clock into relaxed phylogenetics have posited differing, but correlated, rates of mutation along different branches. But filling in these rates requires specifying the topology of the tree—knowing who's related to whom—beforehand. This is often poorly known, and may be the very question phylogeneticists are trying to answer.

Drummond et al. took a different approach. Using a set of artificial DNA sequences generated and mutated to form a rooted tree, they tested five different models of rate variations to determine which most accurately modeled the simulated evolution of this group. The five models included a strict molecular clock, in which mutation rates were the same on all branches at all times, as well as various modifications in which rates were correlated or uncorrelated among the branches. Using the phylogenetic analysis program called BEAST, they found that the most robust model—the one that did best under various starting conditions—was neither the strict molecular clock nor the correlated models, but the “uncorrelated relaxed-clock” models, in which the mutation rates in each branch are allowed to vary but within particular constraints.

They then tested their models in several real sets of data, including viruses, marsupials, plants, bacteria, and yeast. In the plant dataset, which was known to have the most “clock-like” evolution, the strict molecular clock model did best, not surprisingly. But the relaxed-clock model performed best overall among all the datasets, drawing trees that were closest to known relationships with the fewest missteps. And, unlike in the unrooted phylogenic approach, they were able to assign times of divergence to each branch on the tree.

The model developed by the authors promises to bring the very important question of time back into phylogenetic analysis. The ability of the model to create accurate trees may also make it of use even to scientists whose main interests are in understanding phylogenetic relationships, rather than the timing of evolutionary divergence.

